# Stem Cells as a Novel Biomedicine for the Repair of Articular Meniscus: Pharmacology and Applications

**DOI:** 10.3389/fphar.2022.897635

**Published:** 2022-04-26

**Authors:** Qiaoyin Tan, Cuicui Wu, Lei Li, Yijie Liang, Xiaoyong Bai, Weide Shao

**Affiliations:** ^1^ College of Teacher Education, Zhejiang Normal University, Jinhua, China; ^2^ College of Physical Education and Health Sciences, Zhejiang Normal University, Jinhua, China; ^3^ Nova Doctors Group, Hunan Carnation Biotechnology Co., Ltd., Carnation Hospital, Changsha, China

**Keywords:** stem cells, repair and maintenance, articular meniscus, novel biomedicine, biological agent, stem cell therapy

## Introduction

Meniscus is a kind of fibrocartilage composite tissue which plays an important role in the stability, load distribution and shock absorption of the knee joint. In addition, meniscus can absorb the impact and provide nutrition. Meniscus is mainly composed of water, collagen, glycosaminoglycan, etc. Meniscal injury is one of the most common sports injuries. After meniscal injury, traditional repair, suture or meniscectomy are currently used for clinical treatment. However, due to the limited number of blood vessels, it is difficult to heal, and may even lead to knee arthritis and osteoarthritis. Therefore, the field of meniscal in which alternative regenerative medicine is being studied has attracted attention, especially stem cells as the best source of cells to help stimulate the regeneration of natural tissues of meniscal. although only limited relevant research has been conducted at present, mesenchymal stem cells are a very good idea for repairing human joint meniscus injury (Prabhath et al., 2021).

Stem cells have been studied by more and more experts and scholars due to their unique characteristics, such as self-renewal, proliferation and differentiation, immune regulation and plasticity. However, these cells are limited in their clinical application due to their tumorigenesis. However, among the various stem cell types, mesenchymal stem cells (MSCs) have a lower risk of formation due to the teratoma they carry (Huldani et al., 2022)Without any ethical issues, and its readily available sources, have been the subject of most therapeutic application studies. Studies have isolated, cultured and amplified bone marrow mesenchymal stem cells from animals such as sheep and used them in various transformation models for human medical applications (Gugjoo and Amarpal, 2018). There are also many studies that have evaluated MSCs including cartilage, tendon and construct (Gugjoo et al., 2020).

The research summarizes the repair methods of meniscal injury, especially the application of mesenchymal stem cells in the meniscal injury of joint. Many researchers have used a variety of methods to treat meniscal injury with stem cells in animal experiments, to promote the healing of meniscal. Although some results have been achieved, it cannot be widely used in human body, and it is hoped that it can be applied to clinical practice in the near future.

## REPAIR METHOD

### Scaffold and Stem Cell Combination

Meniscal repair techniques have had limited success in previous years due to technical reasons and local vascularization of the meniscus. Then with that development of meniscus repair technique, there is a new technique by suturing the meniscus-capsule complex to the edge of the tibial plateau. This can reduce the meniscal extrusion of the centralizing technique in the treatment of meniscal injury (Ozeki et al., 2021). However, in this way, the scaffold was completely resorbed and no new tissue was formed. Therefore, the combined repair method of scaffolds and stem cells can make up for the defects. Stem cells have strong self-renewal ability and multi-directional differentiation potential, and it is expected to treat meniscus defects by combining with scaffold materials (Arredondo et al., 2021).

Recent advances in stem cell technology have expanded the ability of meniscal stenting. Extracellular matrix of acellular meniscus (MECM) can significantly promote the survival and proliferation of meniscal fibrochondrocytes and increase the *in vitro* expression of type II collagen and proteoglycans. At the same time, the PCL scaffold printed in 3D was used to construct the bionic acellular scaffold with both micro-structure and micro-environment. This cell-free PCL-MECM scaffold demonstrated excellent biocompatibility and yielded good biomechanical properties similar to those of the natural meniscus. Regeneration of the new meniscus in the PCL-MECM scaffold transplanted to the knee and subjected to medial meniscectomy may be a promising approach in rabbit and sheep models. (Guo et al., 2021). In conclusion, the 3D cell printing technique, PCL-MECM scaffold combined with stem cells, helped to generalize the meniscus tissue specificity with respect to the shape and microenvironment of meniscus regeneration (Chae et al., 2021). Composite nanofiber scaffold based on mesenchymal stem cells (MSCs) and tissue engineering constructs (TEC) is helpful to prevent meniscus extrusion and protect cartilage. This meniscus defect is always repaired with fibrocartilage tissue. (Shimomura et al., 2019). Some studies have evaluated the role of polyvinyl alcohol/chitosan (PVA/Ch) scaffolds implanted by adipose-derived mesenchymal stem cells (ASC) and articular chondrocytes (AC) in meniscal regeneration. The results showed that AC/stent group had the best results, followed by AC-ASC/stent group and ASC/stent group (Moradi et al., 2017). Although further research is needed in preclinical application, the results of these studies in clinical meniscal biomaterial repair strategy are promising.

In conclusion, there is a growing recognition of the previous view that menisci cannot be repaired, and that treatment can be performed using repair techniques and bio-enhancing that combine stents with stem cell injection.

### Stem Cell Injection

**FIGURE 1:**
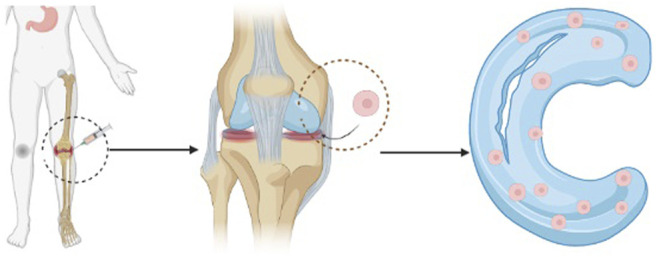
Proces schematic of stem cell injection method.

Many studies have tried to repair meniscus with mesenchymal stem cells. As shown in Figure 1 many people are interested in treating meniscus injury with biological agents such as mesenchymal stem cells and platelet-rich plasma. (Baria et al., 2017). Therapeutic procedures and adjuvant drugs include mechanical stimulation of healing reactions, bone marrow aspirate concentrates, fibrin reinforcement, mesenchymal stem cells, platelet-rich plasma, and other drugs under development used alone or in combination. In particular mesenchymal stem cells, have great research potential for improving and repairing joint meniscus (Mlynarek et al., 2018).

As for the mechanism of stem cells promoting the repair of articular meniscus, there is a tissue engineering strategy based on mesenchymal stem cells (MSC) to test its feasibility for cartilage regeneration. Some studies have derived new stem cell sources from arthroscopic irrigation (AFF-MSCs), which may contain significant amounts of MSCs from synovial and synovial fluid. The experiment prove that wrapping that stem cell with the one-step rapid crosslinking polypeptide DA/HA hydrogel has very encouraging cartilage regeneration potential (Li et al., 2018) Other acellular matrix (DCM) derived from meniscal can induce synovial fluid-derived mesenchymal stem cells (SF-MSCs) to differentiate into meniscal fibrochondrocytes (MFC). (Liang et al., 2018). ECM components from the inner and outer areas of meniscus can promote cartilage formation of mesenchymal stem cells in three-dimensional photo-crosslinked hydrogel. These findings suggest that acellular meniscus ECM has region-specific biological activity. (Rothrauff et al., 2017). In addition to mesenchymal stem cells, allogeneic three-dimensional shaped adipose-derived stem cells (ADSCs) were implanted into the rabbit model with a partially meniscectomy. The results showed that he survived in rabbits and adhered to the defect area, which promoted the histological healing of meniscus (Toratani et al., 2017).

Many studies on repairing meniscal with stem cells show the effectiveness. However, the relationship between MSC life span, persistence at the injured site and therapeutic effect is still unclear (Colbath et al., 2017). The effect of mECM on the response of microencapsulated MSCs and the integrated meniscal repair indicated that mECM hydrogel could be a very promising carrier. MSCs can be transported for long-term repair of meniscal tissue. (Zhong et al., 2020). In addition, ECM hydrogel derived from specific tissues can be injected to provide a bionic environment for cell delivery and realize seamless regeneration of tissue defects. Human bone marrow mesenchymal stem cells (hMSCs) in the meniscus ECM hydrogel facilitate tissue regeneration after injection. It can prevent that development of joint space stenosis and osteoarthritis. Therefore, the study recommends the use of tissue-specific meniscal ECM-derived hydrogels to deliver therapeutic hMSCs for the treatment of meniscal injury. (Yuan et al., 2017). These studies are of great value for exploring its clinical application.

## Conclusion

Mesenchymal stem cells (MSCs) have brought hope for cartilage regeneration and achieved good results in animal models. However, based on the internal and external effectiveness of current animal research, there is no evidence that stem cells can form meniscal similar to primitive human meniscus in human body. Therefore, more in-depth research is needed. In addition, whether the manufacturing process of stem cells is standard, whether the clinical methods of bone tissue engineering can be developed on a large scale, and whether the preclinical research can be effectively transformed into clinical trials need further research.

## Discussions

The research on tissue regeneration technology of stem cells to repair meniscal injury is just started, and has not been widely applied to clinical practice. The therapeutic effect of stem cell repair of meniscus cannot be accurately evaluated. In addition, the high cost of stem cell purification and storage, as well as the need for multiple injections of treatment, are also factors that cannot be fully implemented at present. However, human studies are promising, and MSCs have great potential in the musculoskeletal system. In the next few years, mesenchymal stem cells may play an important role in meniscus repair.
